# Thanking our peer reviewers

**DOI:** 10.1242/bio.017053

**Published:** 2016-02-15

**Authors:** Rachel Hackett, Jordan W. Raff

**Affiliations:** 1 Managing Editor of Biology Open, The Company of Biologists, Bidder Building, Station Road, Cambridge CB24 9LF, UK; 2 Editor-in-Chief of Biology Open and Professor at the Sir William Dunn School of Pathology, University of Oxford, South Parks Road, Oxford OX1 3RE, UK

At the beginning of each year, BiO takes the opportunity to thank its many reviewers for their contribution to the journal. The peer review process is the cornerstone of research publication in the biological sciences. As a publishing company, we are very aware that this vital component of the publication process is done, for free, by scientists worldwide. While we can assist our reviewers in every way possible, by providing online article submission and peer review systems, and having staff trained to support reviewers and answer any questions they might have, the article must be carefully read and the report composed.

Submissions to BiO have steadily increased since its launch in 2011 and doubled since we announced our Impact Factor in June 2015. BiO publishes papers that have been accepted on the basis that they are technically sound and their conclusions are supported by the data shown. We do not require any assessment of the significance, relative importance or impact of a paper. However, the paper should clearly address a non-trivial scientific question. BiO has functionality that allows readers to comment on published papers, in the form of online eLetters, enabling the community to discuss the work.

The advent of the ‘author pays’ model of scientific publication has led to the founding of many ‘predatory’ publishers that charge fees to authors often without providing effective peer review. Initiatives to help authors decide which journals are sound have been implemented. BiO is a member of OASPA (the Open Access Scholarly Publishers Association) and is indexed in the Directory of Open Access Journals (DOAJ), an online directory that lists high-quality, Open Access, peer-reviewed journals. The DOAJ recently made all publishers reapply for inclusion, using more stringent criteria; BiO's reapplication was successful and the journal continues to be listed in the DOAJ. BiO is a member of COPE (http://publicationethics.org/) and also supports the Think. Check. Submit. campaign (thinkchecksubmit.org), which provides a simple checklist researchers can use to assess the credentials of a journal or publisher.

Unbiased independent peer review is at the heart of our publishing decisions, but there is always room for improvement, and we continue to monitor potential developments and the views of our community. In the meantime, we thank our reviewers, who are listed in the supplementary information, for all their hard work and expertise.

